# Introgression and Characterization of a Goatgrass Gene for a High Level of Resistance to Ug99 Stem Rust in Tetraploid Wheat

**DOI:** 10.1534/g3.112.002386

**Published:** 2012-06-01

**Authors:** Daryl L. Klindworth, Zhixia Niu, Shiaoman Chao, Timothy L. Friesen, Yue Jin, Justin D. Faris, Xiwen Cai, Steven S. Xu

**Affiliations:** * USDA–ARS, Northern Crop Science Laboratory, Fargo, North Dakota 58102-2765; † USDA–ARS Cereal Disease Laboratory, St. Paul, Minnesota 55108; ‡ Departments of Plant Sciences, North Dakota State University, Fargo, North Dakota 58108-6050

**Keywords:** wheat, Ug99, *Sr47*, *Aegilops speltoides*, chromosome engineering

## Abstract

The transfer of alien genes to crop plants using chromosome engineering has been attempted infrequently in tetraploid durum wheat (*Triticum turgidum* L. subsp. *durum*). Here, we report a highly efficient approach for the transfer of two genes conferring resistance to stem rust race *Pgt*-TTKSK (Ug99) from goatgrass (*Aegilops speltoides*) to tetraploid wheat. The durum line DAS15, carrying the stem rust resistance gene *Sr47* derived from *Ae. speltoides*, was crossed, and backcrossed, to durum 5D(5B) aneuploids to induce homeologous pairing. After a final cross to ‘Rusty’ durum, allosyndetic recombinants were recovered. The *Ae. speltoides* chromosomal segment carrying *Sr47* was found to have two stem rust resistance genes. One gene conditioning an infection type (IT) 2 was located in the same chromosomal region of 2BS as *Sr39* and was assigned the temporary gene symbol *SrAes7t*. Based on ITs observed on a diverse set of rust races, *SrAes7t* may be the same as *Sr39*. The second gene conditioned an IT 0; and was located on chromosome arm 2BL. This gene retained the symbol *Sr47* because it had a different IT and map location from other stem rust resistance genes derived from *Ae. speltoides*. Allosyndetic recombinant lines carrying each gene on minimal alien chromosomal segments were identified as were molecular markers distinguishing each alien segment. This study demonstrated that chromosome engineering of *Ae. speltoides* segments is feasible in tetraploid wheat. The *Sr47* gene confers high-level and broad spectrum resistance to stem rust and should be very useful in efforts to control TTKSK.

Common wheat (*Triticum aestivum* L., 2*n* = 6*x* = 42, AABBDD) and durum wheat (*T. turgidum* L., subsp. *durum*, 2*n* = 4*x* = 28, AABB) are major food sources (Singh *et al.* 2008). Stem rust (caused by *Puccinia graminis* Pers.:Pers. f.sp. *tritici* Eriks. and Henn.) has historically been one of the most important diseases of these crops (Singh *et al.* 2006). Although resistant cultivars have played a major role in the control of stem rust, the emergence of a new highly virulent race, TTKSK (Ug99), originating in Uganda in 1999, jeopardizes world wheat production (Singh *et al.* 2006, 2011). TTKSK has proven highly virulent, with an estimate of only 5% of Middle East and Southern Asia wheat acreage planted to resistant cultivars in 2005 to 2006 (Singh *et al.* 2008). In North America, the majority of wheat cultivars were susceptible to TTKSK (Jin and Singh 2006) or variants TTKST (Jin *et al.* 2008) and TTTSK (Jin *et al.* 2009). Finding and deploying stem rust resistance genes effective against the Ug99 lineage of races are vital to protecting the world’s wheat supply.

Wild relatives of wheat are important sources of new genes for cultivated wheat. In the past 40 years, numerous desirable genes, including approximately 20 stem rust resistance genes (McIntosh *et al.* 2010; Liu *et al.* 2011; Qi *et al.* 2011), have been transferred into common wheat from its wild relatives by developing wheat-alien species chromosome translocations through chromosome engineering (Friebe *et al.* 1996; Gill *et al.* 2011). Because homologous chromosome pairing in wheat is strictly controlled by *Ph1* (pairing homeologous) on chromosome 5B, translocations between a wheat chromosome and its homeologue in wild species are usually induced using *Ph1* deletion stocks such as a 5D(5B) substitution line or the *ph1b* mutant (Niu *et al.* 2011). Compared with its frequent uses in hexaploid wheat, chromosome engineering has been used sparingly in durum wheat, but the successful transfer of genes for high molecular weight glutenins (Ceolini *et al.* 1996; Joppa *et al.* 1998), disease resistance (Huguet-Robert *et al.* 2001), salt tolerance (Luo *et al.* 1996), and kernel texture (Morris *et al.* 2011) have been documented. One major problem with chromosome engineering in a tetraploid background is poor plant vigor and low fertility of interspecific crosses. This may result from reduced genomic buffering and increased linkage drag as the result of durum wheat having only two genomes (AB), rather than three (ABD) as in common wheat (Ceoloni *et al.* 1996; Gennaro *et al.* 2007).

The durum wheat line DAS15, developed through *ph1b*-induced homeologous recombination by L. R. Joppa, carries the stem rust resistance gene *Sr47* derived from an accession (PI 369590) of *Aegilops speltoides* Tausch (2*n* = 2*x* =14, SS). This gene is highly effective against TTKSK, but it was located on a T2BL-2SL·2SS translocation chromosome in which the distal 2BL segment comprised less than 10% of the long arm, with the remainder of the chromosome originating from *Ae. speltoides* (Faris *et al.* 2008). To make *Sr47* usable in wheat breeding, efforts are needed to reduce the *Ae. speltoides* segment. A set of aneuploids based on Rusty (PI 639869) (Klindworth *et al.* 2006), a near-universal stem rust susceptible genetic stock of durum wheat, has been recently established (Klindworth and Xu 2008). One of these aneuploids is the Rusty 5D(5B) double-monosomic (DM), in which one 5B chromosome has been replaced by chromosome 5D. The objectives of this research were to use the Rusty 5D(5B) aneuploids to reduce the *Ae. speltoides* segment carrying *Sr47* and to test the feasibility of Rusty aneuploids in chromosome engineering of durum wheat.

## Materials and Methods

### Plant materials

DAS15 and Rusty were used for crossing and population development. Rusty is closely related to Line 47-1, differing mainly by plant ideotype and absence of the minor stem rust resistance gene, *SrM* (Klindworth *et al.* 2006). Rusty aneuploids used in this study included the Rusty 2D(2A) disomic substitution (DS), Rusty 2D(2B) DS, and Rusty 5D(5B) DM. Because the Rusty 5D(5B) DS did not yet exist when crossing was initiated, a 47-1 5D(5B) DS (Klindworth *et al.* 2007) was used when a 5D(5B) DS was needed for crosses. Like the Langdon 5D(5B) DS (Joppa and Williams 1988), the 47-1 5D(5B) DS is maintained with a 5B monosome.

### Population development

**Figure 1:**
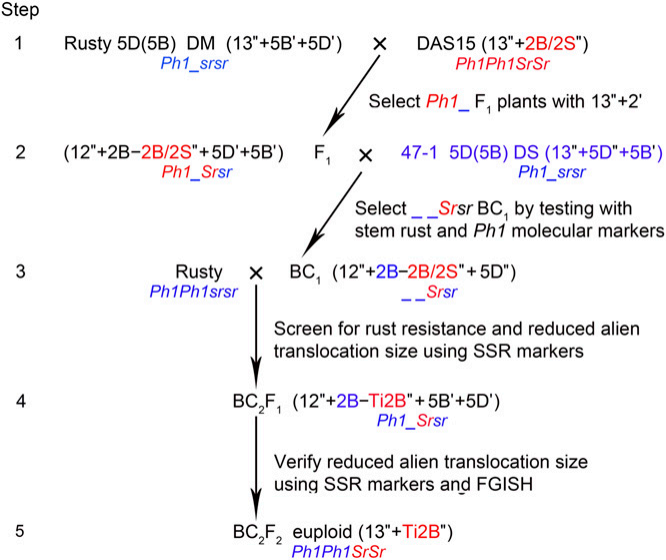
Crossing and selection procedure for production of allosyndetic recombinants of tetraploid wheat carrying stem rust resistance gene *Sr47*. The underline symbol (_) indicates a missing 5B chromosome. Blue indicates genes derived from Rusty or 47-1 aneuploids. Red indicates either genes derived from DAS15 or the new allosyndetic recombinant. Ti2B represents intercallary translocation 2B, 2B-2B/2S″ represents heteromorphic pairings between chromosome 2B and the translocation chromosome 2B/2S, and 2B-Ti2B″ represents heteromorphic pairings between chromosome 2B and the intercallary translocation 2B.

The Rusty 5D(5B) DM was crossed to DAS15 (Figure 1, Step 1), and 20 F_1_ plants were evaluated for chromosome pairing at metaphase I (MI) of meiosis. Seven DM (12″+2B-2B/2S″+5B′+5D′) F_1_ plants with heteromorphic pairing (2B-2B/2S″) between chromosome 2B and the translocation chromosome 2B/2S were selected and backcrossed as females to the 47-1 5D(5B) DS (Figure 1, Step 2). The rationale for using the F_1_ plants as females was to avoid the high male-transmission rate of the 5B monosome, calculated as 90.3% by Joppa and Williams (1988). Similarly, the male transmission of the 5B monosome is greater in 5D(5B) DM than in 5D(5B) DS, thus making it preferable to use the 47-1 5D(5B) DS rather than Rusty 5D(5B) DM as the male in the backcross. The BC_1_F_1_ plants were tested for resistance to *Pgt*-TMLKC using inoculation procedures as mentioned in the section *Rust inoculation procedures*. Resistant BC_1_F_1_ plants were tested for the presence of chromosome 5B using 5BL-specific markers *Xpsr128* and *Xpsr574* (Roberts *et al.* 1999).

In hexaploid wheat, marker *XAWJL3* can be used as a positive amplification check (Roberts *et al.* 1999), but we found that this marker was unreliable in tetraploid wheat (supporting information, Figure S1). Instead, simple sequence repeat (SSR) marker *Xedm80* was used as a positive check (Figure S1 and Figure S2) (Mullan *et al.* 2005). Plants negative for the *Xpsr128* and *Xpsr574* bands were 5D(5B) DS carrying a single 2B/2S translocation chromosome (*i.e.*, 12″+ 2B-2B/2S″+5D″) and they were selected and crossed as males to Rusty to produce a large BC_2_F_1_ population (Figure 1, Step 3). This population was first tested for resistance to TMLKC and then tested for allosyndetic recombination of the *Ae. speltoides* chromatin carrying *Sr47* using capillary electrophoresis as described below (Figure 1, Step 4). Plants exhibiting dissociation were self-pollinated to recover euploid progeny and to select homozygous dissociation lines (Figure 1, Step 5).

### Rust inoculation procedures

Following the procedures of Williams *et al.* (1992), we suspended stem rust urediniospores in nonphytotoxic, paraffinic oil and sprayed on 6- to 8-d-old seedlings. The plants remained in a subdued light mist chamber for 24 hr after inoculation. Seedlings were then moved to a greenhouse at 20 to 23° with supplemental fluorescent light to maintain a 14/10-hr (day/night) photoperiod. Seedlings were classified for stem rust infection type (IT) 12-14 d after inoculation by scoring the infected primary leaf from each plant (Stakman *et al.* 1962; Roelfs and Martens 1988). In this system of notation, 0, fleck (;), 1, or 2 are considered resistant, and 3 or 4 are considered susceptible. For leaves exhibiting combinations of ITs, order indicates predominant types. Minus (^−^), double minus (^=^), and plus (^+^) indicated small, very small, or large pustules within a class.

### Molecular marker analysis

**Figure 2:**
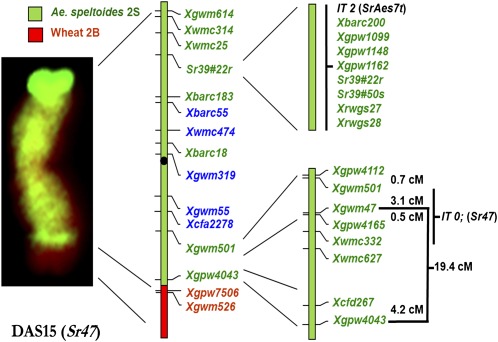
FGISH showing the T2BL-2SL·2SS chromosome in DAS15 and molecular markers used to map allosydetic recombinants. Markers shown in red were monomorphic and therefore located on the wheat segment of the T2BL-2SL·2SS chromosome. Markers in blue were used for capillary electrophoresis of the complete population. Markers in green were used in PAGE to analyze subsets of allosyndetic recombinants identified by capillary electrophoresis. Marker order and distances (cM) generally follow Sourdille *et al.* (2010). Positions of those markers not shown on the Sourdille *et al.* (2010) map were inferred from either Somers *et al.* (2004), Mago *et al.* (2009), Dobrovolskaya *et al.* (2011), or Niu *et al.* (2011). Because of limited published data, order of markers clustered around the IT 2 gene on 2BS/2SS could not be fully determined, and these markers are listed in no particular order.

DNA was extracted from the BC_2_F_1_ population developed above using 96-well plates as described by Niu *et al.* (2011). With the goal of finding SSR markers that are useful for selection of allosyndetic recombinants from the BC_2_F_1_ population, 36 SSR markers spanning the entire 2B/2S chromosome were screened for polymorphism among Rusty, DAS15, Rusty 2D(2A) DS, and Rusty 2D(2B) DS using an ABI 3130xl Genetic Analyzer (Applied Biosystems, Foster City, CA) as described by Tsilo *et al.* (2009). Five codominant markers (*Xgwm55*, *Xgwm319*, *Xwmc474*, *Xbarc55*, and *Xcfa2278*; Figure 2) were found to be suitable for marker-assisted selection in the capillary electrophoresis system (Table S1), and then they were used to genotype the BC_2_F_1_ population as described by Niu *et al.* (2011).

After completing capillary electrophoresis, additional SSR markers located on wheat chromosome 2B or 2S reported by Somers *et al.* (2004), Mago *et al.* (2009), Sourdille *et al.* (2010), Niu *et al.* (2011), and Dobrovolskaya *et al.* (2011) were evaluated for polymorphisms using polyacrylamide gel electrophoresis (PAGE). Four polymorphic markers, *Xgpw4043*, *Xgwm501*, *Sr39#22r*, and *Xgwm614*, were used to evaluate all BC_2_F_1_ allosyndetic recombinants identified by capillary electrophoresis. For all PAGE, polymerase chain reaction products were run on 10 cm mini-gels composed of 8% acrylamide. Electrophoresis was conducted at 150 V for 40 min if expected products were less than 150 bp. For expected products larger than 150 bp, electrophoresis was conducted for 50-55 min with the exception of *Xrwgs27*, *Xrwgs28*, and *Xrwgs29*, for which electrophoresis was extended to 135 min. Polymerase chain reaction products were stained with 2X GelRed, and gels were visualized with UV light and photographed.

In progeny evaluations, homozygous rust-resistant BC_2_F_2_ plants were identified either through marker analysis or stem rust testing. For marker analysis, homozygous IT 0; and IT 2 plants were identified using markers *Xgpw4043* or *Sr39#50s*, respectively. For rust tests, homozygous BC_2_F_2_ plants were identified by BC_2_F_3_ progeny tests. DNA from BC_2_F_2_ plants identified as homozygous by either selection method was included in additional marker tests.

### Tests for segregation distortion and validation of markers

Five allosyndetic recombinant lines were selected to test for segregation distortion. Progeny from plants known to be heterozygous for the translocated segment were tested with race TMLKC and classified as resistant or susceptible. For each family, plants were tested with appropriate SSR markers and classified as homozygous resistant, heterozygous, or susceptible. Data were tested for goodness of fit to a 1:2:1 ratio using χ^2^ analysis. Marker validation was tested on Rusty, LMPG6, and the set of eight durum and 32 common wheat cultivars described by Niu *et al.* (2011). LMPG6 is a common wheat line from Canada with spring growth habit that is near-universally susceptible to stem rust (Knott 1990).

### Florescent genomic *in situ* hybridization (FGISH) and measurement of translocation breakpoints and fraction lengths

FGISH was used to detect *Ae. speltoides* segments in the allosyndetic recombinant lines by using the genomic DNA of *Ae. speltoides* PI 369590 and common wheat cultivar ‘Chinese Spring’ as probe and blocking DNA, respectively. FGISH was performed using the protocol described by Yu *et al.* (2010). The fraction lengths (FL) of translocation breakpoints relative to chromosomal length (Endo and Gill 1996; Friebe *et al.* 1996) in nine intercalary translocation (Ti) 2BL-2SL-2BL·2BS lines were measured. The distal and proximal FL values were measured as the distance from the 2BL telomere to the distal or proximal breakpoints divided by the chromosomal length. The *Ae. speltoides* segment FL value was the proximal minus the distal FL value. Lengths were measured in 17 to 21 good-quality mitotic metaphase cells per line. Data were analyzed as a completely randomized design using the SAS GLM procedure (SAS Institute 2004), and means were separated by least significant difference.

## Results

### Development and selection of allosyndetic recombinants

There were 218 BC_1_ (Figure 1, Step 2) plants having the pedigree Rusty 5D(5B)/DAS15//47-1 5D(5B) tested for resistance to race TMLKC, and these plants segregated 89 susceptible to 129 resistant. This segregation did not fit a 1:1 ratio (χ^2^ = 7.34, *P* = 0.007), and because 47-1 5D(5B) was the male parent of the cross, this result suggested there was minor segregation distortion through female gametes. Resistant plants were tested with chromosome 5BL-specific markers *Xpsr128* and *Xpsr574* (Figure S2). There were 52 BC_1_ plants that did not carry chromosome 5B as indicated by the failure to amplify the *Xpsr128* and *Xpsr574* alleles on 5B. These plants were 5D(5B) DS, which had 28 chromosomes with pairing configurations of 12″+2B-2S/2B″+5D″. Because of their lack of chromosome 5B, homeologous pairing would occur in these 52 BC_1_ plants, and they were crossed as males to Rusty to produce 1086 BC_2_F_1_ seeds for use in selection of recombinant lines.

**Figure 3:**
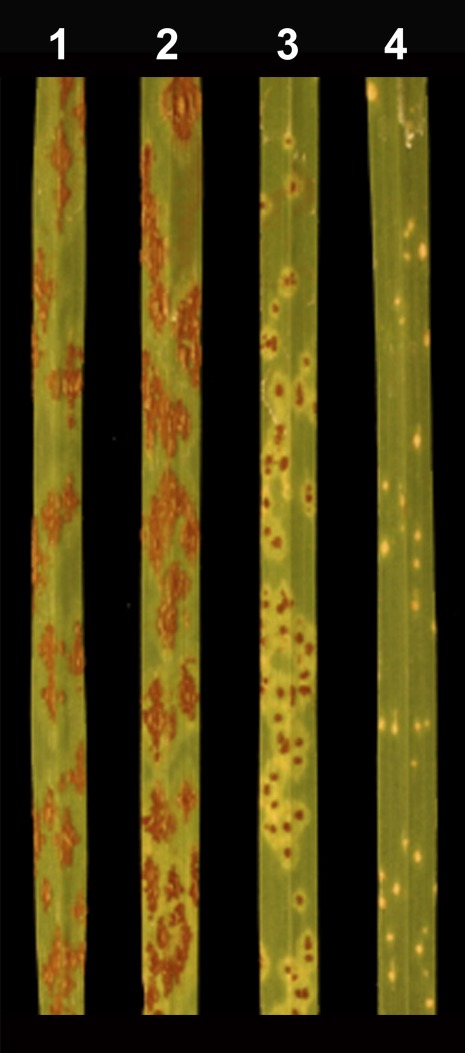
Stem rust ITs observed in (1) Rusty and (2−4) three BC_2_F_1_ plants of Rusty/3/Rusty 5D(5B)/DAS15//47-1 5D(5B). Plants 1 and 2 exhibit IT 34 (susceptible), whereas plant 3 has IT 2 and plant 4 has a IT fleck (0;).

The 1086 BC_2_F_1_ plants were tested with TMLKC. There were 893 resistant and 193 susceptible plants, which did not fit a 1:1 segregation ratio (χ^2^ = 451.2, *P* < 0.001). Because Rusty was the female parent of the cross, the results indicated male gametes had strong segregation distortion favoring transmission of the *Ae. speltoides* segment. The resistant plants comprised two distinct ITs. There were 856 plants that had an IT 0; and 37 plants that had IT 2 (Figure 3), indicating that the *Ae. speltoides* segment in DAS15 carried two stem rust resistance genes. The two genes are here temporarily referred to as the IT 0; and IT 2 gene. The 1086 BC_2_F_1_ plants were genotyped with the five SSR markers, *Xgwm55* (Figure S3), *Xgwm319*, *Xwmc474*, *Xbarc55*, and *Xcfa2278*, using capillary electrophoresis. The marker analysis and stem rust test identified 81 allosyndetic recombinant plants (Table S2) that are summarized in Table 1. Forty-two of the plants had IT 0; and 37 plants had IT 2. Among the IT 2 plants, 32 retained the *Ae. speltoides* alleles for all five SSR markers. These 32 plants were identified as new allosyndetic recombinants because of the absence of the IT 0; gene. Two of the 81 allosyndetic recombinant plants were susceptible to TMLKC (Table 1 and Table S2). For both plants, the wheat alleles at four of the five SSR loci were replaced by the *Ae. speltoides* alleles.

**Table 1:** Summary of haplotypes for nine SSR markers observed in the BC_2_F_1_ generation of Rusty/3/Rusty 5D(5B)/DAS15//47-1 5D(5B) allosyndetic recombinants

IT	*Xgpw* *4043*	*Xgwm* *501*	*Xcfa* *2278*	*Xgwm* *55*	*Xgwm* *319*	*Xwmc* *474*	*Xbarc* *55*	*Sr39* *#22r*	*Xgwm* *614*	No. of Plants
0;	S	W	W	W	W	W	W	W	W	7
0;	S	W	W	W	W	W	W	W	S	2
0;	W	S	W	W	W	W	W	W	W	5
0;	S	S	W	W	W	W	W	W	W	23
0;	S	S	W	W	W	W	W	W	S	1
0;	S	S	W	W	W	S	S	S	S	1
0;	S	S	W	S	W	W	W	W	W	1
0;	S	S	S	S	S	W	W	W	W	1
0;	W	S	S	S	S	W	W	W	W	1
Total										42
2	S	S	S	S	S	S	S	S	S	1
2	W	S	S	S	S	S	S	S	S	5
2	W	S	S	S	S	S	S	S	W	1
2	S	W	S	S	S	S	S	S	S	2
2	W	W	S	S	S	S	S	S	S	20
2	W	W	S	S	S	S	S	S	W	1
2	S	W	S	S	S	S	S	S	W	2
2	S	W	W	W	W	S	S	S	S	1
2	W	W	W	W	W	S	S	S	S	1
2	S	W	W	W	W	W	S	S	S	1
2	S	W	W	W	W	W	W	S	W	1
2	W	W	W	W	W	W	W	S	W	1
Total										37
34	S	S	S	S	S	S	W	W	S	1
34	W	S	S	S	S	S	W	W	W	1
Total										2

Markers are listed in order from most distal on 2BL (left) to most distal on 2BS (right) as suggested by maps of Sourdille *et al.* (2010) and Mago *et al.* (2009). S, *Ae. speltoides* allele; W, wheat allele.

The 81 allosyndetic recombinant plants were further genotyped by PAGE with four additional SSR markers (*Xgpw4043*, *Xgwm501*, *Sr39#22r*, and *Xgwm614*), with two located in each arm and each located distal to the markers tested by capillary electrophoresis (Table 1 and Table S2). When all nine SSR markers were considered, the IT 0; and IT 2 recombinant plants comprised 9 and 12 haplotypes, respectively (Table 1). For the IT 0; gene, all 42 allosyndetic recombinants retained the *Ae. speltoides* allele for either *Xgpw4043* alone (9 plants), *Xgwm501* alone (6 plants), or both markers (27 plants). For the remaining 7 markers, no marker retained the *Ae. speltoides* allele in more than 4 of 42 plants (Table 1). This result indicated that the IT 0; gene in DAS15 was located near *Xgpw4043* and *Xgwm501* on 2BL. Among the 37 IT 2 allosyndetic recombinants (Table 1), the two having the shortest *Ae. speltoides* segment retained the *Sr39#22r* allele from *Ae. speltoides*. The only *Ae. speltoides* allele retained in all 37 plants was the *Sr39#22r* allele, indicating that the IT 2 gene was located near *Sr39#22r* in 2BS.

The 81 BC_2_F_1_ plants had chromosome pairing configurations of 12″+ 2B-2B/2S″+5B′+5D′ at MI. Both the 2B-2B/2S heteromorphic bivalent and the DM condition reduce plant fertility. These plants produced on average 13.6 seeds per plant, with nine plants being sterile and one plant (0696) producing 209 seeds (Table S2). On the basis of marker analysis or stem rust testing on BC_2_F_2_ progenies, 14 and seven BC_2_F_2_ plants that were homozygous for ITs 0; and 2, respectively, were selected for additional marker analysis. In selecting markers to test on IT 0; plants, only markers proximal to *Xgpw7506* were studied because *Xgpw7506* was located in the wheat segment of the original translocation chromosome in DAS15 (Figure 2 and Figure S4). Polymorphisms for *Xgpw4112*, *Xgwm501*, and *Xgwm47* were attributable to allele-specific amplification failure, which could be expressed as either a difference in staining intensity or as absence of the amplicon (Figure S5). Line 0406 carried wheat alleles for the four most distal markers (Table 2, Figure S5). Seven lines, including Line 0010, carried wheat alleles for the two most proximal markers. The combination of these results indicated that the IT 0; gene was located between markers *Xgwm501* and *Xwmc332* (Table 2). The homozygous IT 2 Line 0797 carried the shortest interstitial translocation, with wheat alleles at both the *Xbarc183* and *Xwmc25* loci (Table 3 and Figure S6). The IT 2 gene lies between these two loci, along with eight markers whose map order was not determined (Figure 2).

**Table 2:** Haplotypes for seven SSR markers in 14 homozygous Ti2BL-2SL-2BL·2BS allosyndetic recombinants carrying the IT 0; (fleck) gene from DAS15

Line	*Xgpw* *4043*	*Xcfd* *267*	*Xwmc* *627*	*Xwmc* *332*	*Xgpw* *4165*	*Xgwm* *47*	*Xgwm* *501*	*Xgpw* *4112*
0010	S	?	S	S	S	S	W	W
0143	S	S	S	S	S	S	W	W
0198	S	S	S	S	S	S	S	S
0225	S	S	S	S	S	S	W	W
0406	W	W	W	W	S	S	S	S
0439	S	S	S	S	S	S	S	S
0466	S	W	S	S	S	S	W	W
0623	S	?	S	S	S	S	S	S
0696	S	S	S	S	S	S	S	S
0717	S	W	S	S	S	S	W	W
0735	S	S	S	S	S	S	S	S
0790	S	S	S	S	S	S	W	W
0801	W	S	W	S	S	S	S	S
0804	S	W	S	S	S	S	W	W

Markers are listed in order from most distal (left) to most proximal (right) on 2BL as suggested by maps of Sourdille *et al.* (2010) and Somers *et al.* (2004). S, *Ae. speltoides* allele; ?, unknown; W, wheat allele.

**Table 3:** Haplotypes for 17 SSR markers in seven allosyndetic recombinants carrying the IT 2 gene from DAS15

Line	*Xgpw4043*	*Xgwm501*	*Xgwm319*	*Xbarc18*	*Xbarc55*	*Xbarc183*	*Sr39#50s*	*Sr39#22r*	*Xrwgs27*	*Xrwgs28*	*Xbarc200*	*Xgpw1148*	*Xgpw1162*	*Xgpw1099*	*Xwmc25*	*Xwmc314*	*Xgwm614*
0111	S	W	W	W	S	S	S	S	S	S	S	S	S	S	S	S	S
0151	W	S	S	W	S	S	S	S	S	S	S	S	S	S	S	W	S
0744	W	W	W	W	W	S	S	S	S	S	S	S	S	S	S	W	W
0797	S	W	W	W	W	W	S	S	S	S	S	S	S	S	W	W	W
0902	S	W	S	S	S	S	S	S	S	S	S	S	S	S	S	S	W
1009	S	W	W	W	S	S	S	S	S	S	S	S	S	S	S	S	S
1043	W	S	S	S	S	S	S	S	S	S	S	S	S	S	S	W	W

Markers are generally listed in order from most distal on 2BL (left) to most distal on 2BS (right) as suggested by maps of Xue *et al.* (2008), Mago *et al.* (2009), Sourdille *et al.* (2010), and Dobrovolskaya *et al.* (2011). However, for the eight markers detecting only *Ae. speltoides* chromatin in all seven lines, marker order could not be determined from this study. S, *Ae. speltoides* allele; W, wheat allele.

### FGISH analysis and measurements of Ti2BL-2SL-2BL·2BS fraction lengths

**Figure 4:**
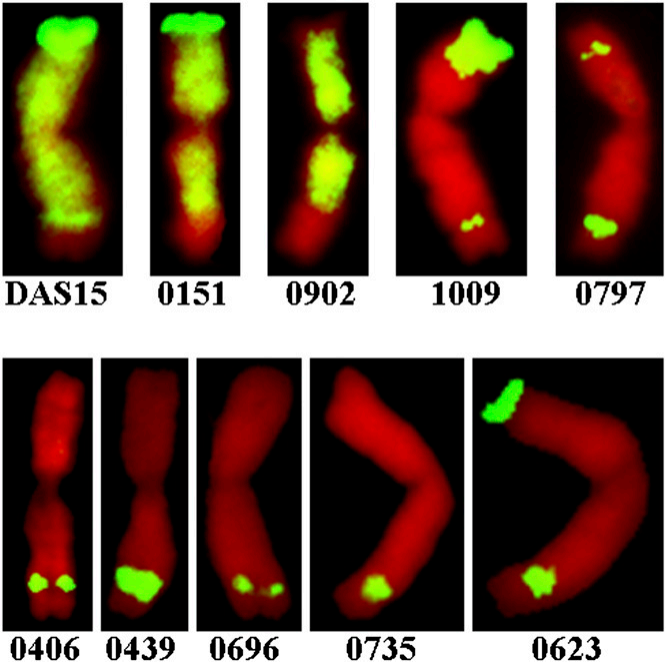
FGISH results for DAS15 and nine allosyndetic recombinants carrying either the IT 0; or IT 2 gene from DAS15. Green fluorescence is *Ae. speltoides* chromatin labeled with fluorescein isotiocyanate-conjugated avidin (FITC-avidin). Red fluorescence is wheat chromatin labeled with propidium iodide. Lines 0151, 0902, 1009, and 0797 carry the IT 2 gene on 2BS. Lines 0406, 0439, 0696, 0735, and 0623 carry the IT 0; gene on 2BL.

Fourteen IT 0; and five IT 2 allosyndetic recombinants were analyzed by FGISH, and micro-photographs for five lines having IT 0; and four lines having IT 2, are shown in Figure 4. All IT 2 lines retained an *Ae. speltoides* segment in chromosome arm 2BS, with Line 0797 retaining the shortest segment. Lines 0151 and 0902 were IT 2 lines having only small reductions of *Ae. speltoides* chromatin in the subtelomeric region of 2BL (Figure 4). The IT 0; gene must be located in this small deleted segment; therefore, the FGISH confirmed that the IT 0; gene was located near the break-point of the original 2B/2S translocation chromosome in DAS15. All IT 0; lines retained an *Ae. speltoides* chromosomal segment in the subtelomeric region of 2BL.

An interesting feature revealed by the FGISH analysis was that some lines had recombination events in both chromosome arms (Figure 4). This finding agreed with results from the marker analysis. For example, Lines 1009 and 0797 retained the *Ae. speltoides* chromatin around *Sr39#22r* and *Sr39#50s* in 2BS and also retained the *Ae. speltoides* allele for *Xgpw4043* in 2BL (Figure 4; Table 3).

The physical positions of the translocation breakpoints in nine Ti2BL-2SL-2BL·2BS allosyndetic recombinant lines were determined by measuring the distance from the 2BL telomere to the distal and proximal breakpoints. The nine lines carried relatively small *Ae. speltoides* segments (Table 4). The distal FL breakpoints indicated that wheat chromatin comprised between 0.0900 and 0.1238 of the 2BL telomere and subtelomeric region (Table 4). Lines 0406, 0439, and 0801 had high distal breakpoint values, and this was in general agreement with the molecular marker analysis, which showed that, in this region, lines 0406 and 0801 had the wheat allele at *Xgpw4043* (Table 2). Furthermore, the low proximal breakpoint values of Lines 0717, 0790, and 0804 (Table 4) was in agreement with these lines carrying wheat alleles at *Xgwm501* and *Xgpw4112* (Table 2).

**Table 4:** FL of the wheat segments and the *Ae. speltoides* segments in nine Ti2BL-2SL-2BL·2BS translocation chromosomes

	FL Value*^a^*		
Dissociation Line	Proximal Breakpoint*^b^*	Wheat Segment (Distal Breakpoint)*^c^*	*Ae. speltoides* Segment*^d^*
0406	0.1916 AB	0.1235 A	0.0680 BCD
0439	0.2031 A	0.1238 A	0.0793 A
0623 (plant 1)	0.1886 AB	0.1084 C	0.0802 A
0623 (plant 2)	0.1733 CD	0.1084 C	0.0649 CD
0696	0.1812 BC	0.1098 BC	0.0714 ABC
0717	0.1717 CD	0.1111 BC	0.0606 D
0735	0.1728 CD	0.1038 CD	0.0690 BCD
0790	0.1648 D	0.0900 E	0.0748 AB
0801	0.1968 A	0.1200 AB	0.0767 AB
0804	0.1653 D	0.0942 DE	0.0711 ABC
DAS15	−	0.0675 F	0.9325
Mean	0.1814	0.1055	0.0719*^e^*
LSD (*P* = 0.05)	0.0149	0.0109	0.0097*^e^*

FL, fraction length; LSD, least square diference.

a Means followed by the same letter were not significantly different as determined by LSD.

b The length from the 2BL telomere to the proximal breakpoint divided by the whole chromosome length.

c The length from the 2BL telomere to the distal breakpoint divided by the whole chromosome length.

d Calculated as the proximal breakpoint minus the distal breakpoint.

e Means and LSD for the size of the *Ae. speltoides* segment were calculated excluding the data from DAS15.

### Segregation distortion and stem rust resistance of selected translocation lines and validation of markers

Lines 0406, 0696, and 0717, carrying the IT 0; gene, and lines 0744 and 0797 carrying the IT 2 gene, were tested for segregation of resistance and to confirm that the markers could be used for marker-assisted selection (Table S3). Lines 0744 and 0797 were selected because they carried the IT 2 gene on the shortest *Ae. speltoides* segments. Lines 0406, 0696, and 0717 were selected because they carried the IT 0; gene on short, but slightly different, *Ae. speltoides* segments (Table 2). Progeny from a known heterozygous plant for each of the five lines were tested with stem rust and markers. Results indicated significant segregation distortion in all populations except Line 0406 (Table S3). Segregation distortion resulted in selection against the alien segment in the two lines carrying the IT 2 gene, Lines 0744 and 0797, where only 5.0% and 3.4% of plants were homozygous resistant, respectively. This finding was reversed in Lines 0696 and 0717, where an excess of resistant plants was observed, indicating preferential transmission of the *Ae. speltoides* segment.

Two IT 0; lines (0406 and 0696) and one IT 2 line (1009) with the shortest *Ae. speltoides* segments were tested with race TTKSK and 13 North American races (Table 5). Lines 0406 and 0696 had only minor differences from each other on the 14 races. ITs observed on 0406 and 0696 ranged from 0; to 0;1^−^ on the North American races, but when tested with TTKSK, an IT of ;2^−^ was observed on both lines. Although this was a greater IT than observed on DAS15, it still provided a good level of resistance to TTKSK. Line 1009 having IT 2 was compared with line RWG1 (Niu *et al.* 2011), which carries *Sr39*. The results indicated highly similar ITs of Line 1009 and RWG1. Some minor difference could be attributed to genetic background or ploidy level. We concluded that over the 14 races in the test, the ITs conditioned by the genes in Line 1009 and RWG1 did not differ.

**Table 5:** ITs of *Sr47* allosyndetic recombinant lines and RWG1 carrying *Sr39* when tested with 14 races of stem rust*^a^*

Line	TTKSK	TPMKC	TPPKC	TMLKC	TCMJC	THTSC	RHTSC	RTQQC	QTHJC	QFCSC	QCCJB	MCCFC	HKHJC	HPGJC
Rusty	4	43	34	43	32	34	33^+^	3	43	43	43	43	43	43
DAS15	;	0;	0;	0;1^-^	0;1^=^	0;1^=^	0;1^=^	0;	0;1^-^	0;	0;	0;	0;	0;
RWG 35 (0406) (*Sr47*)	;2^-^	0;	0;	0;1^-^	0;1^=^	0;1^=^	0;1^=^	0;	0;1^-^	0;	0;	0;	0;	0;
RWG 36 (0696) (*Sr47*)	;2^-^	0;	0;	0;1^-^	0;1^=^	0;1^=^	0;1^=^	0;	0;	0;	0;	0;	0;	0;1^=^
Line 1009 (*SrAes7t*)	2^-^	2^-^	2^-^	2^-^	12^-^	12^-^	12^-^	2^-^	12^-^	1^+^	2^=^	12^-^	21	12^-^
RWG1 (*Sr39*)	2^-^	2^-^	2^-^	2^-^	12^-^	12^-^	12^-^	1^+^	12^-^	1^+^2^-^	2^-^	12	21	12^-^

IT, infection types.

a ITs follow Stakman *et al.* (1962) where 0, fleck (;), 1, or 2, are considered resistant, and 3 or 4 are considered susceptible. For leaves exhibiting combinations of ITs, order indicates predominant types; *e.g.*, IT 34 is predominantly IT 3 with decreasing amounts of IT 4. Minus (^−^), double minus (^=^), and plus (^+^) indicated small, very small, or large pustules within a class.

**Figure 5:**
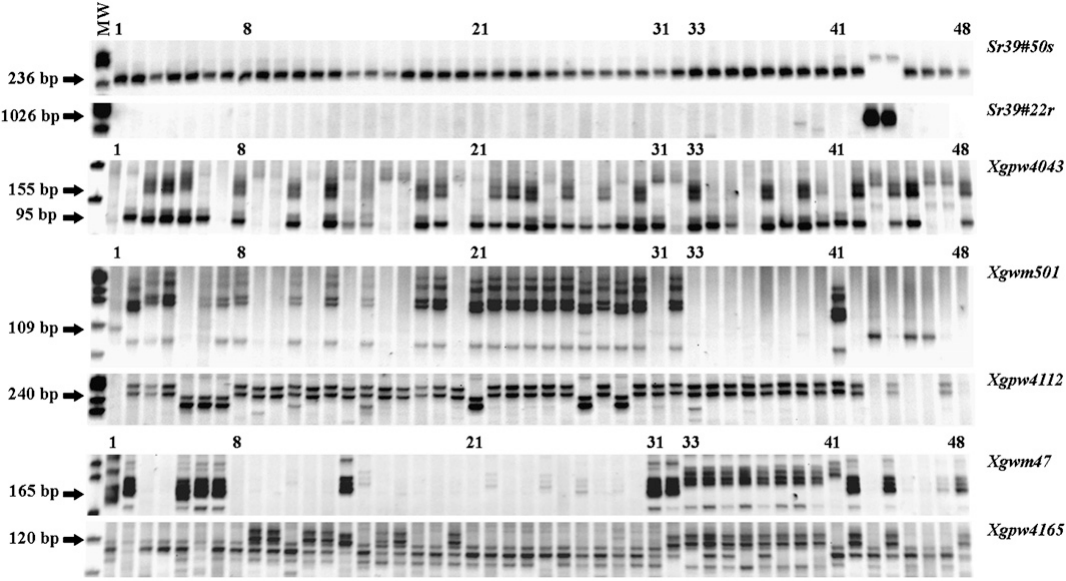
Validation of seven molecular markers on 40 common and durum wheat cultivars. Lanes 1−7 are Chinese common wheat cultivars. Lanes 8−32 are North American common wheat cultivars. Lanes 33−40 are North American durum cultivars. Lanes 41−48 are checks. Lane IDs are MW) molecular weight marker, (1) Jimai22, (2) Yangmai16, (3) Shanrong1, (4) Shanrong3, (5) Jinan17, (6) Jinan177, (7) Zhengmai9023, (8) Amidon, (9) Howard, (10) Alsen, (11) Grandin, (12) Glenn, (13) Faller, (14) Glupro, (15) Ernest, (16) Steele, (17) Reeder, (18) Mott, (19) Kulm, (20) Parshall, (21) Granger, (22) Brick, (23) Russ, (24) Briggs, (25) Traverse, (26) Sabin, (27) Oklee, (28) Ulen, (29) Ada, (30) Tom, (31) Newton, (32) IL06-14262, (33) Divide, (34) Ben, (35) Tioga, (36) Grenora, (37) Lebsock, (38) Monroe, (39) Alkabo, (40) Mountrail, (41) LMPG6, (42) Rusty, (43) DAS15, (44) Line 0744, (45) Line 0406, (46) Line 0696, (47) Line 0717, and (48) Rusty.

Seven markers were tested on a set of 40 diverse common and durum wheat cultivars (Figure 5). Markers *Sr39#50s* and *Sr39#22r* were linked to the IT 2 gene on 2BS. *Sr39#50s* is a codominant marker that amplified a 268-bp fragment from *Ae. speltoides* and a 236-bp fragment in all 40 cultivars (Figure 5 and Figure S6) and is the preferred marker. Dominant marker *Sr39#22r* amplified a 1026-bp fragment from *Ae. speltoides* that was absent in all 40 cultivars (Figure 5). The five remaining markers were all linked to the IT 0; gene on 2BL. Among these markers, the dominant marker *Xgpw4112* produced a null allele from the *Ae. speltoides* and Chinese cultivar Jimai22, but it amplified fragments in 39 of the 40 cultivars (Figure 5). For *Xgpw4112*, there was polymorphism among cultivars. The dominant marker *Xgwm501* amplified a 109-bp fragment from *Ae. speltoides* that was absent in 39 cultivars (Figure 5). In Rusty, marker *Xgpw4043* resulted from the amplification of 95 and 155 bp fragments (Figure S5). Polymorphism was observed among cultivars for marker *Xgpw4043*, including cultivars in which only the 155 bp fragment was absent, and cultivars null for both fragments (Figure 5). However, the 95-bp fragment (Figure S5) was observed in 30 of the 40 cultivars (Figure 5), making *Xgpw4043* useful for most cultivars. Markers *Xgwm47* and *Xgpw4165* amplified fragments in all eight durum cultivars, but each produced fragments in only eight common wheat cultivars (Figure 5). In summary, the five markers associated with the IT 0; gene produced good amplification of fragments with durum wheat, but breeders will need to carefully match markers with cultivars to transfer the IT 0; gene to common wheat cultivars.

## Discussion

Chromosome engineering of tetraploid wheat has been attempted less frequently than in hexaploid wheat. This has partially been attributable to the fact that more research is conducted on hexaploid than on tetraploid wheat. Another factor has been lower genomic buffering of tetraploid wheat, which results in decreased plant fertility and lower recovery rates of allosyndetic recombinants. We monitored seed fertility of DM plants in this experiment to determine whether low seed set would prevent the use of durum 5D(5B) aneuploids in chromosome engineering. Although the seed set was very low, with a mean of only 13.6 seeds per plant, this was sufficient to conclude that chromosome engineering in tetraploid wheat is feasible. However, the efficiency of chromosome engineering in tetraploid wheat would be more dependent on the strategies for development of allosyndetic recombinants than in hexaploid wheat. Both F_2_ (Qi *et al.* 2007) and backcross (Marais *et al.* 2010; Niu *et al.* 2011) populations have been successfully used to develop *ph1b*-induced allosyndetic recombinants in hexaploid wheat. The use of a backcross in the present experiment may have been advantageous over an F_2_ population in improving seed fertility of progenies derived from the hybrids with 5D(5B) aneuploids and in hastening the transfer of allosyndetic recombinants to a euploid background.

Niu *et al.* (2011) used a single SSR marker, *Xgwm319*, to identify allosyndetic recombinants for *Sr39*. In the present experiment, we used *Xgwm319* and four other SSR markers in our initial screening. These five markers were clustered in the pericentronomic regions of 2BS and 2BL (Figure 2). The results indicated that using relatively few markers in the proximal regions of the 2B chromosome was highly effective in recovering allosyndetic recombinants. For example, if we had relied only on marker *Xgwm319*, we would have recovered 40 of the 42 allosyndetic recombinants that carried the IT 0; gene and would have recovered all five IT 2 lines having the shortest *Ae. speltoides* chromatin (Table 2). This was caused by lower allosyndetic recombination within the pericentronomic regions as compared with subtelomeric or telomeric regions. This result agreed with the conclusions of Lukaszewski (1995), who found that *ph1b*-induced homeologous recombination of chromosomes 7A of wheat and 7S of *Ae. speltoides* was concentrated in the distal regions and absent near the centromere. We found several double recombinant events, and in this regard, our study differs from Lukaszewski (1995) and Lukaszewski *et al.* (2004). Our recovery of double recombinant events on a single chromosome may reflect the higher homology of chromosomes 2B and 2S as compared to the rye (*Secale cereale* L.) 2R chromosome.

Segregation distortion is a common feature in wheat-*Ae. speltoides* crosses (Zhang and Dvořák 1990). We observed segregation distortion in our initial crosses and also within four of five selected recombinants having short *Ae. speltoides* segments. In an intervarietal wheat map, Xue *et al.* (2008) observed 15 wheat segments carrying segregation distortion loci, including one on 2BL and 2BS, with the 2BL locus being centered on *Xgwm47*. In another intervarietal wheat mapping study, Paillard *et al.* (2003) noted that segregation distortion in chromosome 2B was greater than any other chromosome. In addition to segregation distortion genes, gametocidal genes are present on chromosome 2S of *Ae. speltoides* (Tsujimoto and Tsunewaki 1988). Our results support the conclusion that *Sd* and/or *Gc* genes play a large role in the difficulty of recovery of S-/B-genome allosyndetic recombinants.

We found that the T2BL-2SL·2SS chromosome in DAS15 actually carried two stem rust resistance genes. The IT 0; gene was located in a 2SL chromosome segment and lying between *Xgwm501* and *Xwmc332*. The size of this interval has been estimated as little as 3 cM on the Wheat Composite-2004 map (Graingenes), to 8 cM in the Wheat Consensus map (Somers *et al.* 2004). Therefore, the IT 0; gene may be tightly linked to *Xgwm47* and *Xgpw4165*. The stem rust resistance gene *Sr9a* was mapped to only 0.9 cM distal to *Xgwm47* (Tsilo *et al.* 2007). On the basis of their similar map positions, it is possible that the IT 0; gene is homoeoallelic to *Sr9*. The IT 2 gene was located to a Ti2BL·2BS-2SS-2BS chromosome and found to lie in the interval between *Xbarc183* and *Xwmc25*. We found eight molecular markers that map to this region, including *Xgpw1148*, which has been mapped to bin 2BS3-0.75-0.84 (Sourdille *et al.* 2010). This is the same region shown by Niu *et al.* (2011) to carry *Sr39*. In our stem rust test, we found that, for all races, the IT 2 gene in Line 1009 conditioned similar ITs to *Sr39* in RWG1. The *Sr39* gene is tightly linked to marker *Sr39#22r* (Mago *et al.* 2009; Niu *et al.* 2011). On the basis of Line 0797 (Table 3), the IT 2 gene from DAS15 is also tightly linked to *Sr39#22r*. All available data suggest that the IT 2 gene could be *Sr39*. However, it is possible that in the future the genes may be shown to be nonallelic, or a stem rust race may be identified that can differentiate the IT 2 gene of DAS15 from *Sr39*.

We selected five allosyndetic recombinants (0406, 0696, 0717, 0744, and 0797) as breeding lines and assigned RWG (RWG 35−RWG 39) designations for each line (Table S3). Several molecular markers that detected the *Ae. speltoides* chromatin were associated with the two stem rust resistance genes. Ideally, markers should be closely linked and codominant. The codominant marker *Sr39#50s* was found to be compatible with all 40 cultivars in our validation tests, making it a good marker for detection of the IT 2 gene. Among markers detecting the IT 0; gene, only *Xgpw4043* was codominant. However, the validation test indicated *Xgpw4043* was compatible with only 30 of the 40 cultivars, and it cannot be used with Line 0406 (Table 2). For Line 0406, selection must be based on marker *Xgwm501* combined with *Xgwm47*, *Xgpw4112*, or *Xgpw4165*. It should also be noted that if the IT 0; gene is homoeoallelic to *Sr9*, then based on the map of Sourdille *et al.* (2010), it is located approximately 20 cM from *Xgpw4043*. Although recombination between wheat and *Ae. speltoides* chromosomal segments is rare in the presence of *Ph1*, it has been noted to occur (Yu *et al.* 2010); and there is a chance of recombination between the IT 0; locus and the *Xgpw4043* locus.

The gene symbol *Sr47* was previously assigned with the assumption that DAS15 carried a single gene for stem rust resistance (Faris *et al.* 2008; McIntosh *et al.* 2010). Our finding that DAS15 carried two genes necessitates assigning the *Sr47* symbol to only one of the two. The ITs observed on the new allosyndetic recombinant lines carrying the IT 0; gene show the greatest similarity to the ITs observed in DAS15 and are dissimilar from ITs produced by *Sr39* (Table 5) or *Sr32* derived from *Ae. speltoides* (Faris *et al.* 2008). In addition, the IT 2 gene may not differ from *Sr39*. The IT 0; gene therefore retained the symbol *Sr47* and the IT 2 gene is assigned the temporary gene symbol *SrAes7t*. Genes conditioning IT 0; to TTKSK are rare, especially for those of wheat origin, although such genes have been identified in *Ae. tauschii* Cosson (Rouse *et al.* 2011) and other relatives of wheat (Jin *et al.* 2007). Thus, with its high level of resistance to TTKSK, *Sr47* should be a valuable new gene for the improvement of stem rust resistance in wheat.

## Supplementary Material

Supporting Information
