# Supplementary material for: Introgression and Characterization of a Goatgrass Gene for a High Level of Resistance to Ug99 Stem Rust in Tetraploid Wheat
Source: G3 (Bethesda). 2012 Jun 1;2(6):665–73. doi: 10.1534/g3.112.002386 (PMC3362296; doi:10.1534/g3.112.002386)
Supplement: Supporting Information [file supp_2_6_665__index.html]

Supporting Information 

# Introgression and Characterization of a Goatgrass Gene for a High Level of Resistance to Ug99 Stem Rust in Tetraploid Wheat

## Supporting Information for Klindworth *et al*, 2012

**Files in this Data Supplement:**

- Supporting Information - Figures S1-S6 and Tables S1-S3 (PDF, 2 MB)
- Figure S1 - Monoplex and duplex tests determining suitability of *XAWJL3* and *Xedm80* as positive controls in multiplex tests of *Ph1* specific markers (PDF, 204 KB)
- Figure S2 - Detection of tetraploid wheat plants lacking *Ph1* (nullisomic 5B) by use of 5B specific markers, *Xpsr128* and *Xpsr574* (PDF, 314 KB)
- Figure S3 - Capillary electropherograms for SSR marker *Xgwm55* in Rusty, DAS15, and aneuploid lines Rusty 2D(2A) and Rusty 2D(2B) (PDF, 206 KB)
- Figure S4 - Electrophoregrams showings tests of two SSR markers on parental and aneuploid durum lines (PDF, 410 KB)
- Figure S5 - Fourteen homozgyous IT 0; lines tested with eight molecular markers that locate to wheat chromosome arm 2BL (PDF, 393 KB)
- Figure S6 - Seven homozgyous IT 2 lines tested with 13 molecular markers that locate to wheat chromosome arm 2BS (PDF, 692 KB)
- Table S1 - Fragment sizes of microsatellite (SSR) amplicons used to test for allosyndetic recombination of *Sr47* (PDF, 66 KB)
- Table S2 - Allosyndetic recombinants in the BC2F1 generation of Rusty/3/Rusty 5D(5B)/DAS15//47-1 5D(5B) classified for nine SSR markers and for plant vigor and fertility a (PDF, 97 KB)
- Table S3 - Segregation for rust resistance and marker alleles among progeny of heterozygous plants of five allosyndetic recombinant lines (PDF, 58 KB)
