# Supplementary material for: Introgression and Characterization of a Goatgrass Gene for a High Level of Resistance to Ug99 Stem Rust in Tetraploid Wheat
Source: G3 (Bethesda). 2012 Jun 1;2(6):665–73. doi: 10.1534/g3.112.002386 (PMC3362296; doi:10.1534/g3.112.002386)
Supplement: Supporting Information [file supp_2.6.665_002386SI.pdf]

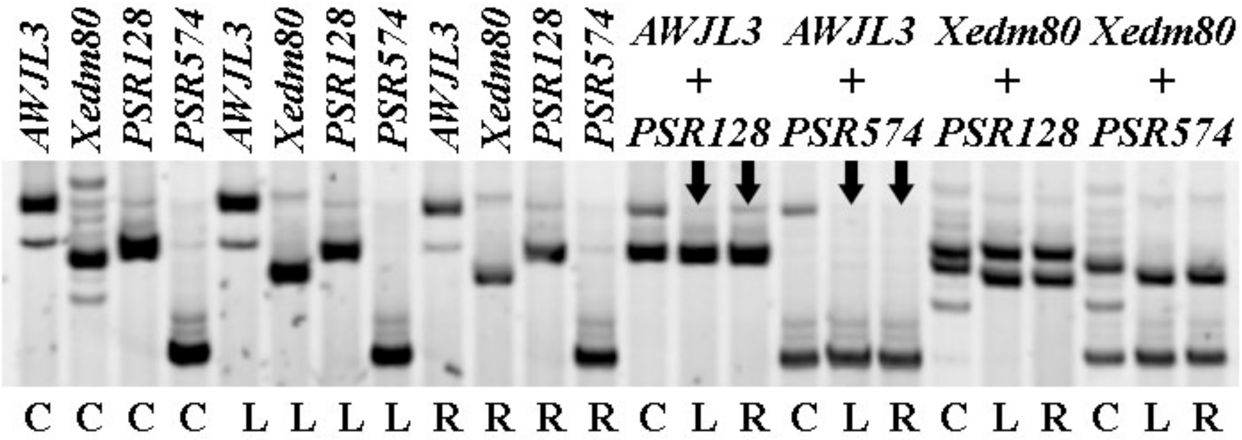

Figure S1 Monoplex and duplex tests determining suitability of *XAWJL3* and *Xedm80* as positive controls in multiplex tests of *Ph1* specific markers. C = Chinese Spring, L = Langdon, R = Rusty. Monoplex and duplex tests are on left and right, respectively. Monoplex tests indicate *XAWJL3* amplicons in all three genetic stocks. In duplex tests, *XAWJL3* was amplified normally in Chinese Spring. Arrows point to absence of *XAWJL3* amplicons due to poor or complete lack of amplification in duplex tests of Langdon and Rusty. *Xedm80* was amplified normally in duplex tests for all genetic stocks indicating that it is better positive check when testing for *Ph1* in Langdon or Rusty. Note the polymorphism in amplicon size for *Xedm80* on Chinese Spring as compared to Langdon and Rusty.

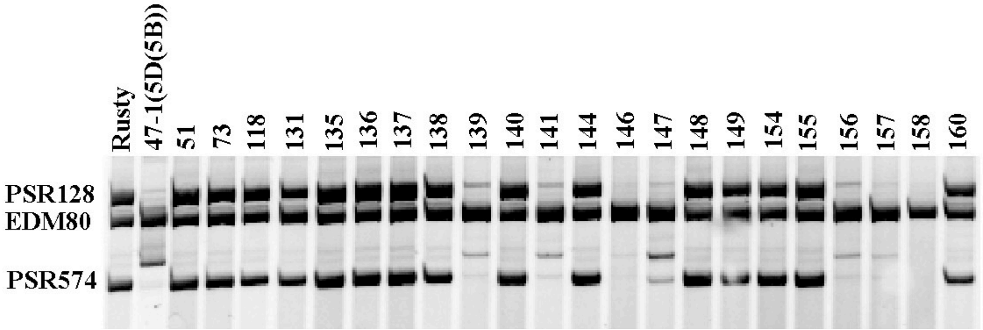

Figure S2 Detection of tetraploid wheat plants lacking *Ph1* (nullisomic 5B) by use of 5B specific markers, *Xpsr128* and *Xpsr574*. Marker *Xedm80* served as a positive check for amplification. Plants lacking the *Xpsr128* and *Xpsr574* amplicons were nullisomic for chromosome 5B.

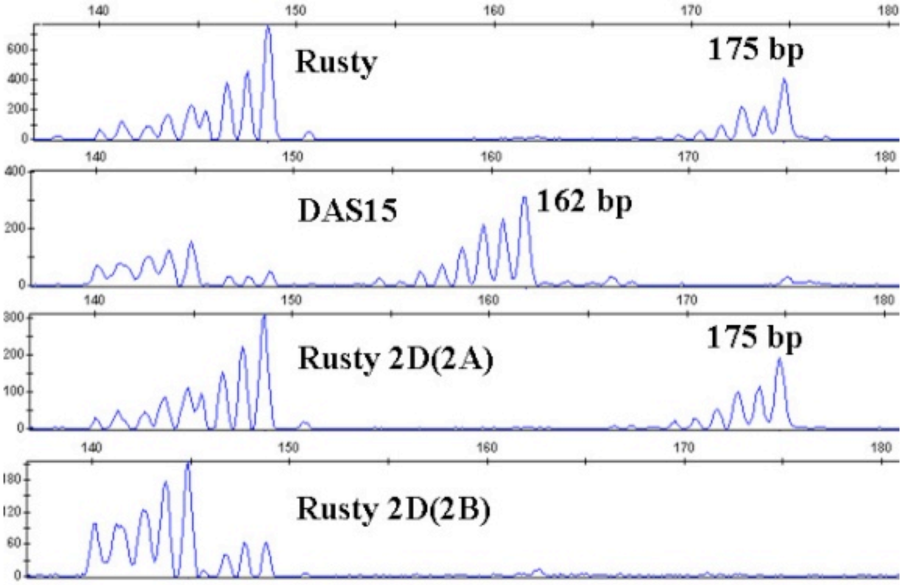

Figure S3 Capillary electropherograms for SSR marker *Xgwm55* in Rusty, DAS15, and aneuploid lines Rusty 2D(2A) and Rusty 2D(2B). The aneuploid lines were used to show the amplicons were derived from loci located on chromosome 2B. Amplicon size includes a 19-bp M13 primer tail. DAS15 amplified a 162 bp fragment, while Rusty amplified a 175 bp fragment from chromosome 2B.

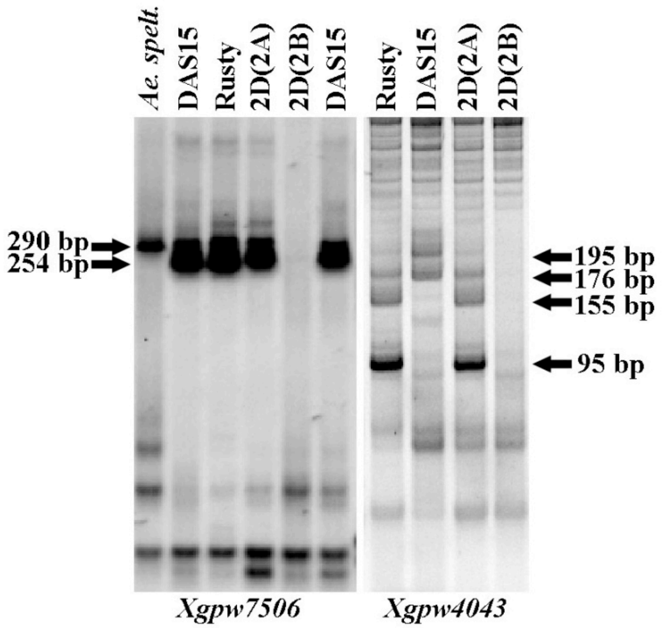

Figure S4 Electrophoregrams showings tests of two SSR markers on parental and aneuploid durum lines. *Ae. spelt.* is *Aegilops speltoides* accession PI 369590, the parental line of DAS15. Rusty and DAS15 did not differ for the 254 bp band from *Xgpw7506*, suggesting DAS15 carried wheat chromatin at the *Xgpw7506* locus. In contrast, polymorphism was observed at the *Xgpw4043* locus located 23 cM proximal of *Xgpw7506* (Sourdille *et al*. 2010). Therefore, the 2SL/2BL interchange in DAS15 was located between these two markers.

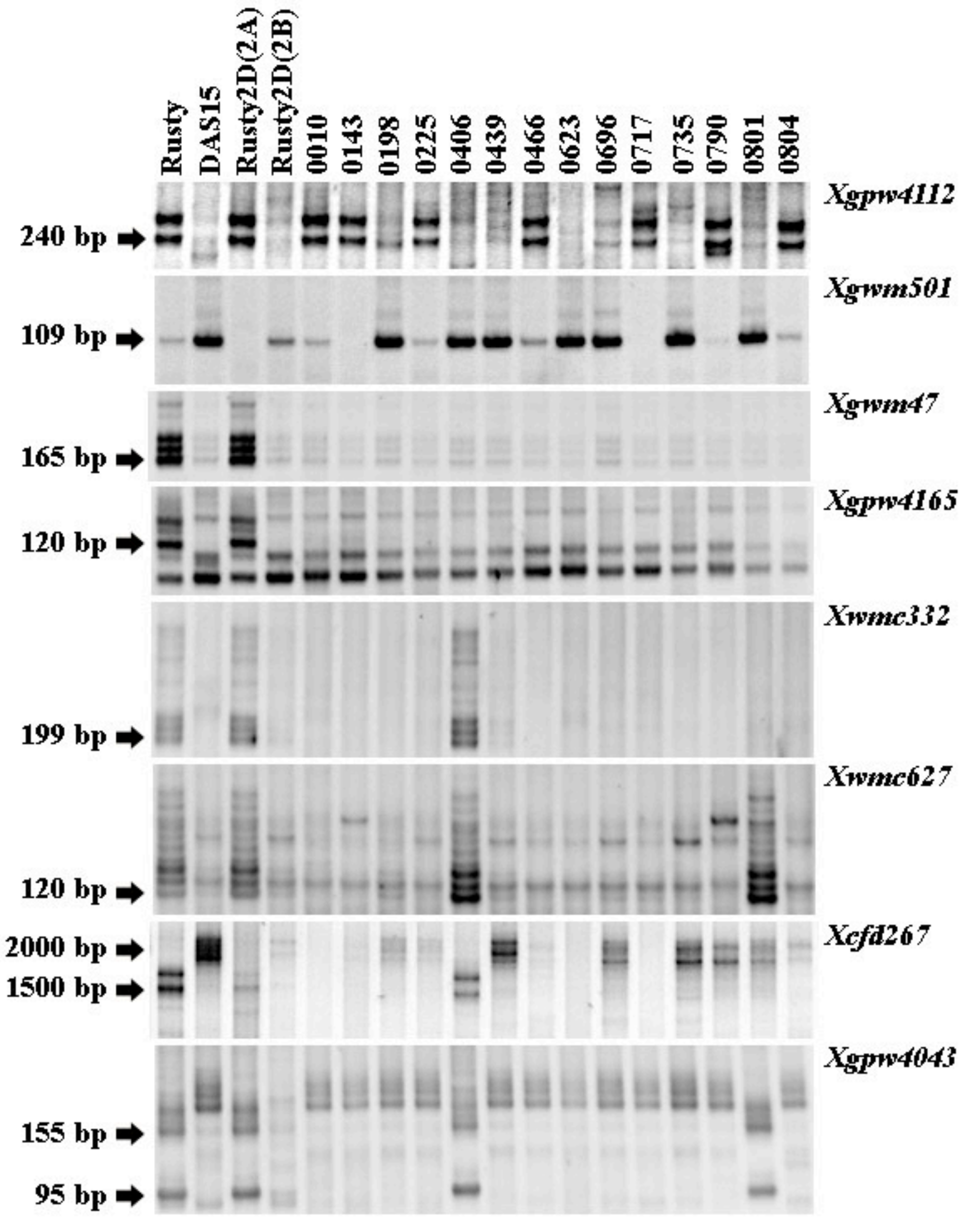

Figure S5 Fourteen homozgyous IT 0; lines tested with eight molecular markers that locate to wheat chromosome arm 2BL. Amplification of the *Xcfd267* bands was inconsistent, possibly due to the large amplicon size.

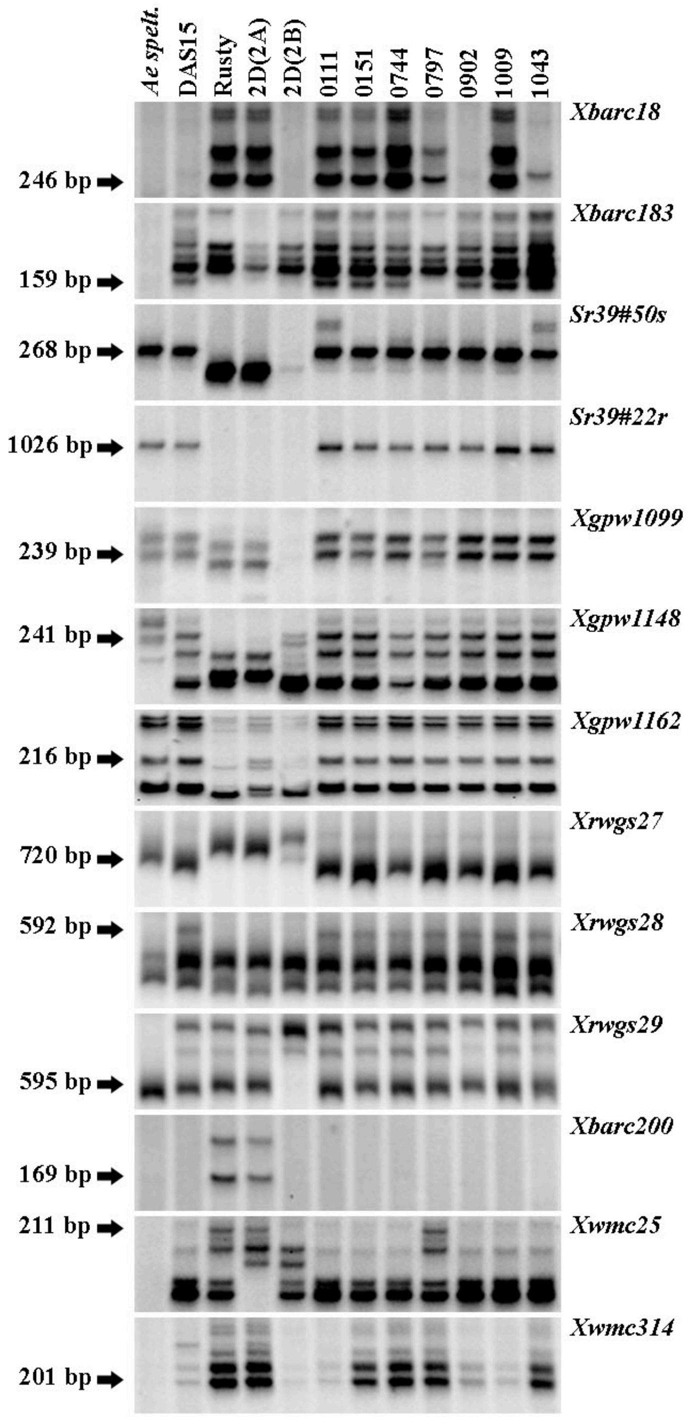

Figure S6 Seven homozgyous IT 2 lines tested with 13 molecular markers that locate to wheat chromosome arm 2BS. *Ae. spelt.* is *Aegilops speltoides* accession PI 369590, the parental line of DAS15. Marker *Xrwgs29* produced a 595 bp band located to wheat chromosome 2B that was either monomorphic or could not be differentiated under mini-gel electrophoretic conditions.

Table S1 Fragment sizes of microsatellite (SSR) amplicons used to test for allosyndetic recombination of *Sr47*

SSR Marker	No. of Tests	Fragment size (bp) [a]	
		Rusty	DAS15
Xcfa2278	2	142	152
Xgwm55	1	175	162
Xgwm319	1	193	182
Xwmc474	2	161-163[b]	172-174[b]
Xbarc55	2	154-159[b]	136

[a] Fragment size includes a 19-bp M13 primer tail
[b] Variable fragment sizes were due to minor differences observed in some check lines

Table S2 Allosyndetic recombinants in the BC_2F_1 generation of Rusty/3/Rusty 5D(5B)/DAS15//47-1 5D(5B) classified for nine SSR markers and for plant vigor and fertility [a]

	Stem Rust IT	Plant No.	*Xgpw 4043*	*Xgwm 501*	*Xcfa 2278*	*Xgwm 55*	*Xgwm 319*	*Xwmc 474*	*Xbarc 55*	*Sr39 #22r*	*Xgwm 614*	No. of spikes	No. of seeds
1	0;	0010	S	W	W	W	W	W	W	W	S	8	23
2	0;	0011	S	S	W	W	W	W	W	W	W	5	0
3	0;	0040	S	S	W	W	W	W	W	W	W	8	26
4	0;	0058	W	S	W	W	W	W	W	W	W	11	28
5	0;	0077	S	S	W	W	W	W	W	W	W	8	18
6	0;	0078	S	S	W	W	W	W	W	W	W	4	15
7	0;	0114	S	S	W	W	W	W	W	W	W	5	22
8	0;	0142	S	S	W	W	W	W	W	W	W	7	19
9	0;	0143	S	W	W	W	W	W	W	W	W	7	8
10	0;	0198	S	S	W	W	W	S	S	S	S	11	47
11	0;	0224	S	S	W	W	W	W	W	W	W	6	15
12	0;	0225	S	W	W	W	W	W	W	W	S	8	7
13	0;	0240	S	S	W	W	W	W	W	W	W	11	25
14	0;	0307	W	S	W	W	W	W	W	W	W	10	18
15	0;	0364	W	S	W	W	W	W	W	W	W	7	15
16	0;	0406	W	S	W	W	W	W	W	W	W	10	15
17	0;	0439	S	S	W	W	W	W	W	W	W	14	21
18	0;	0448	S	S	W	W	W	W	W	W	W	7	7
19	0;	0458	S	S	S	S	S	W	W	W	W	3	0
20	0;	0466	S	W	W	W	W	W	W	W	W	6	11
21	0;	0605	S	S	W	W	W	W	?	W	W	11	35
22	0;	0606	S	S	W	W	W	W	W	W	W	15	27
23	0;	0613	S	W	W	W	W	W	W	W	W	11	12
24	0;	0623	S	S	W	W	W	W	W	W	S	8	15

25	0;	0662	S	S	W	S	W	W	?	W	W	5	0
26	0;	0696	S	S	W	W	W	W	W	W	W	15	209
27	0;	0715	S	S	W	W	W	W	W	W	W	10	33
28	0;	0717	S	W	W	?	W	W	W	W	W	6	18
29	0;	0730	S	W	W	W	W	W	W	W	W	4	3
30	0;	0735	S	S	W	W	W	W	W	W	W	3	4
31	0;	0759	S	S	W	W	?	W	W	W	W	11	14
32	0;	0773	S	S	W	W	W	W	W	W	W	4	13
33	0;	0775	S	S	W	W	W	W	W	?	W	5	18
34	0;	0777	W	S	S	S	S	W	W	?	W	11	0
35	0;	0790	S	W	W	W	W	W	W	W	W	10	18
36	0;	0801	W	S	W	W	W	W	W	W	W	10	16
37	0;	0804	S	W	W	W	W	W	W	W	W	6	14
38	0;	0812	S	S	W	W	W	W	W	W	W	7	11
39	0;	0906	S	S	W	W	W	W	W	W	W	16	70
40	0;	0958	S	S	W	W	W	W	W	W	W	10	8
41	0;	0972	S	S	W	W	W	W	W	W	W	12	16
42	0;	1042	S	S	W	W	W	W	W	W	W	6	5
1	2	0111	S	W	W	W	W	W	S	S	S	10	7
2	2	0744	W	W	W	W	W	W	W	S	W	8	10
3	2	0797	S	W	W	W	W	W	W	S	W	3	10
4	2	1002	W	W	W	W	W	S	S	S	S	6	12
5	2	1009	S	W	W	W	W	S	S	S	S	6	13
6	2	0016	W	W	S	S	S	S	S	S	S	9	18
7	2	0120	W	S	S	S	S	S	S	S	S	6	2
8	2	0150	W	W	S	S	S	S	S	S	S	6	2
9	2	0151	W	S	S	S	S	S	S	S	S	8	13

10	2	0242	W	S	S	S	S	S	S	S	S	4	9
11	2	0311	W	S	S	S	S	S	S	S	S	9	0
12	2	0340	W	W	S	S	S	S	S	S	S	9	3
13	2	0361	W	W	S	S	S	S	S	S	S	8	4
14	2	0380	S	W	S	S	S	S	S	S	S	3	6
15	2	0384	W	W	S	S	S	S	S	S	S	6	1
16	2	0387	W	W	S	S	S	S	S	S	S	3	0
17	2	0431	S	S	S	S	S	S	S	S	S	5	9
18	2	0438	W	W	S	S	S	S	S	S	S	5	0
19	2	0556	S	W	S	S	S	S	S	S	S	9	0
20	2	0608	W	W	S	S	S	S	S	S	S	3	1
21	2	0615	W	W	S	S	S	S	S	S	W	9	5
22	2	0679	W	W	S	S	S	S	S	S	S	7	4
23	2	0866	W	W	S	S	S	S	S	S	S	3	7
24	2	0884	W	S	S	S	S	S	S	S	S	2	1
25	2	0901	W	W	S	S	S	S	S	S	S	3	7
26	2	0902	S	W	S	S	S	S	S	S	W	5	12
27	2	0974	W	W	S	S	S	S	S	S	S	13	3
28	2	0975	W	W	S	S	S	S	S	S	S	7	7
29	2	0979	W	W	S	S	S	S	S	S	S	8	9
30	2	0989	W	W	S	S	S	S	S	S	S	9	2
31	2	1014	W	W	S	S	S	S	S	S	S	2	3
32	2	1041	W	W	S	S	S	S	S	S	S	5	2
33	2	1043	W	S	S	S	S	S	S	S	W	3	5
34	2	1051	S	W	S	S	S	S	S	S	W	7	3
35	2	1105	W	?	S	S	S	S	S	S	S	4	1
36	2	1138	W	?	S	S	S	S	S	S	S	6	0

37	2	1174	W	W	S	S	S	S	S	S	S	7	2
1	34	0414	S	S	S	S	S	S	W	W	S	5	6
2	34	0946	W	S	S	S	S	S	W	W	W	1	1

[a] The plants not listed in this table were 191 stem rust susceptible that retained wheat chromatin for the five markers studied by capillary electrophoresis, and 814 IT 0; plants that retained *Ae. speltoides* chromatin for the five markers studied by capillary electrophoresis

W = wheat allele, S = *Ae. speltoides* allele, question mark (?) indicates that the plant could not be scored for that marker

In summarizing this data for Table 1, markers scored as question mark (?) were assigned the value of the proximal and distal markers if they were identical. For example, plant 0605 was assigned W for *Xbarc55* because *Xwmc474* and *Sr39#22r* had both been scored as W

Table S3 Segregation for rust resistance and marker alleles among progeny of heterozygous plants of five allosyndetic recombinant lines

						χ^2 (1:2:1)		Freq.
Line	IT	Marker	HR	HetR	S	value	*p* (1:2:1)	HR
RWG 35 (0406)	0;	*Xgwm501* & *Xgwm47*	25	40	14	3.1	0.215	0.316
RWG 36 (0696)	0;	*Xgpw4043*	39	39	8	23.1	<0.001	0.453
RWG 37 (0717)	0;	*Xgpw4043*	27	38	8	10.0	0.007	0.370
RWG 38 (0744)	2	*Sr39#50s*	3	24	33	32.4	<0.001	0.050
RWG 39 (0797)	2	*Sr39#50s*	3	51	33	23.3	<0.001	0.034

HR = Homozygous resistant, HetR = Heterozygous resistant, S = Homozygous susceptible. Stem rust resistant plants were classified as homozygous or heterozygous based on molecular marker analysis
