# Supplementary material for: Introgression and Characterization of a Goatgrass Gene for a High Level of Resistance to Ug99 Stem Rust in Tetraploid Wheat
Source: G3 (Bethesda). 2012 Jun 1;2(6):665–73. doi: 10.1534/g3.112.002386 (PMC3362296; doi:10.1534/g3.112.002386)
Supplement: Supporting Information [file supp_2.6.665_FigureS1.pdf]

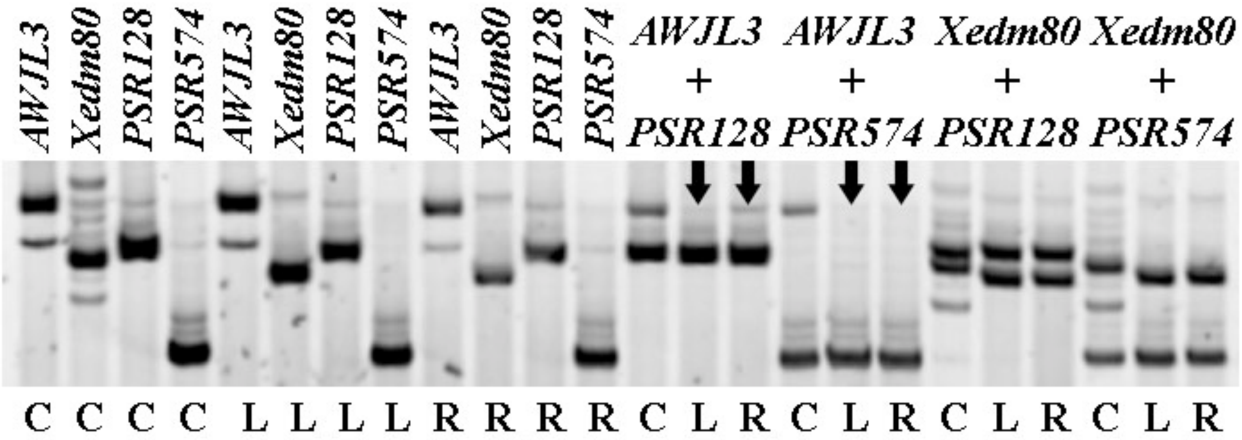

Figure S1 Monoplex and duplex tests determining suitability of *XAWJL3* and *Xedm80* as positive controls in multiplex tests of *Ph1* specific markers. C = Chinese Spring, L = Langdon, R = Rusty. Monoplex and duplex tests are on left and right, respectively. Monoplex tests indicate *XAWJL3* amplicons in all three genetic stocks. In duplex tests, *XAWJL3* was amplified normally in Chinese Spring. Arrows point to absence of *XAWJL3* amplicons due to poor or complete lack of amplification in duplex tests of Langdon and Rusty. *Xedm80* was amplified normally in duplex tests for all genetic stocks indicating that it is better positive check when testing for *Ph1* in Langdon or Rusty. Note the polymorphism in amplicon size for *Xedm80* on Chinese Spring as compared to Langdon and Rusty.
