# Supplementary material for: Introgression and Characterization of a Goatgrass Gene for a High Level of Resistance to Ug99 Stem Rust in Tetraploid Wheat
Source: G3 (Bethesda). 2012 Jun 1;2(6):665–73. doi: 10.1534/g3.112.002386 (PMC3362296; doi:10.1534/g3.112.002386)
Supplement: Supporting Information [file supp_2.6.665_FigureS2.pdf]

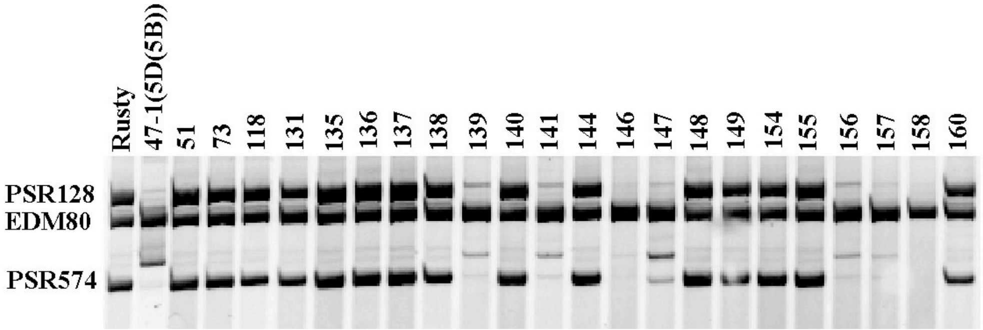

Figure S2 Detection of tetraploid wheat plants lacking *Ph1* (nullisomic 5B) by use of 5B specific markers, *Xpsr128* and *Xpsr574*. Marker *Xedm80* served as a positive check for amplification. Plants lacking the *Xpsr128* and *Xpsr574* amplicons were nullisomic for chromosome 5B.
