# Supplementary material for: Introgression and Characterization of a Goatgrass Gene for a High Level of Resistance to Ug99 Stem Rust in Tetraploid Wheat
Source: G3 (Bethesda). 2012 Jun 1;2(6):665–73. doi: 10.1534/g3.112.002386 (PMC3362296; doi:10.1534/g3.112.002386)
Supplement: Supporting Information [file supp_2.6.665_FigureS3.pdf]

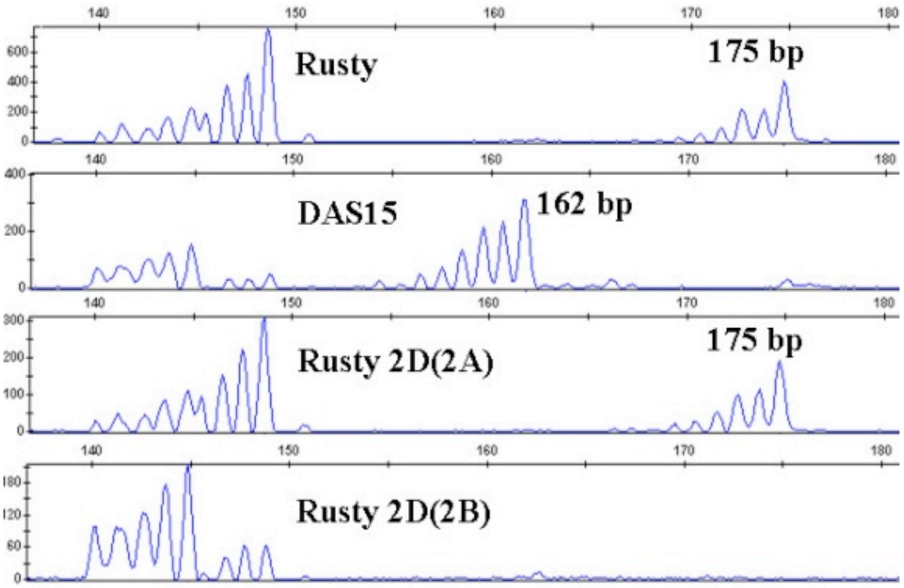

Figure S3 Capillary electropherograms for SSR marker *Xgwm55* in Rusty, DAS15, and aneuploid lines Rusty 2D(2A) and Rusty 2D(2B). The aneuploid lines were used to show the amplicons were derived from loci located on chromosome 2B. Amplicon size includes a 19-bp M13 primer tail. DAS15 amplified a 162 bp fragment, while Rusty amplified a 175 bp fragment from chromosome 2B.
