# Supplementary material for: Introgression and Characterization of a Goatgrass Gene for a High Level of Resistance to Ug99 Stem Rust in Tetraploid Wheat
Source: G3 (Bethesda). 2012 Jun 1;2(6):665–73. doi: 10.1534/g3.112.002386 (PMC3362296; doi:10.1534/g3.112.002386)
Supplement: Supporting Information [file supp_2.6.665_FigureS4.pdf]

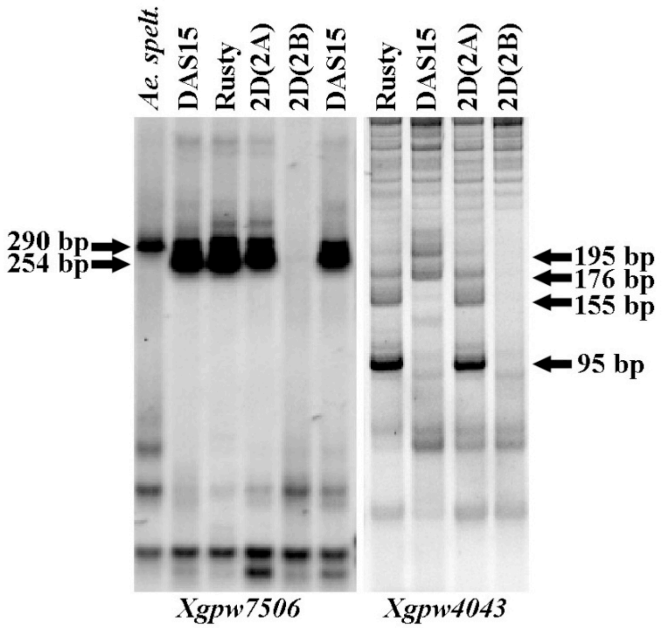

Figure S4 Electrophoregrams showings tests of two SSR markers on parental and aneuploid durum lines. *Ae. spelt.* is *Aegilops speltoides* accession PI 369590, the parental line of DAS15. Rusty and DAS15 did not differ for the 254 bp band from *Xgpw7506*, suggesting DAS15 carried wheat chromatin at the *Xgpw7506* locus. In contrast, polymorphism was observed at the *Xgpw4043* locus located 23 cM proximal of *Xgpw7506* (Sourdille *et al*. 2010). Therefore, the 2SL/2BL interchange in DAS15 was located between these two markers.
