# Supplementary material for: Introgression and Characterization of a Goatgrass Gene for a High Level of Resistance to Ug99 Stem Rust in Tetraploid Wheat
Source: G3 (Bethesda). 2012 Jun 1;2(6):665–73. doi: 10.1534/g3.112.002386 (PMC3362296; doi:10.1534/g3.112.002386)
Supplement: Supporting Information [file supp_2.6.665_FigureS5.pdf]

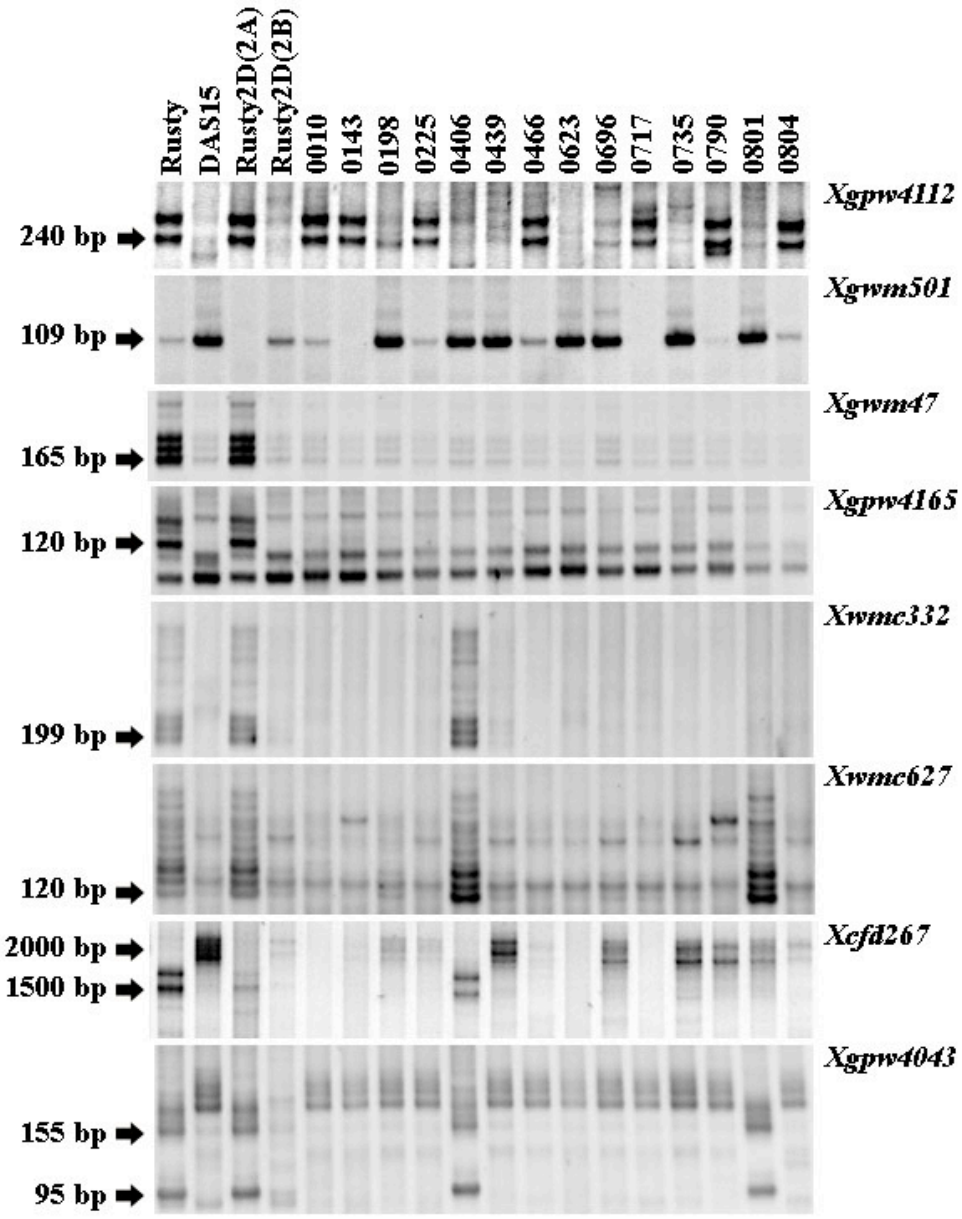

Figure S5 Fourteen homozgyous IT 0; lines tested with eight molecular markers that locate to wheat chromosome arm 2BL. Amplification of the *Xcfd267* bands was inconsistent, possibly due to the large amplicon size.
