# Supplementary material for: Introgression and Characterization of a Goatgrass Gene for a High Level of Resistance to Ug99 Stem Rust in Tetraploid Wheat
Source: G3 (Bethesda). 2012 Jun 1;2(6):665–73. doi: 10.1534/g3.112.002386 (PMC3362296; doi:10.1534/g3.112.002386)
Supplement: Supporting Information [file supp_2.6.665_FigureS6.pdf]

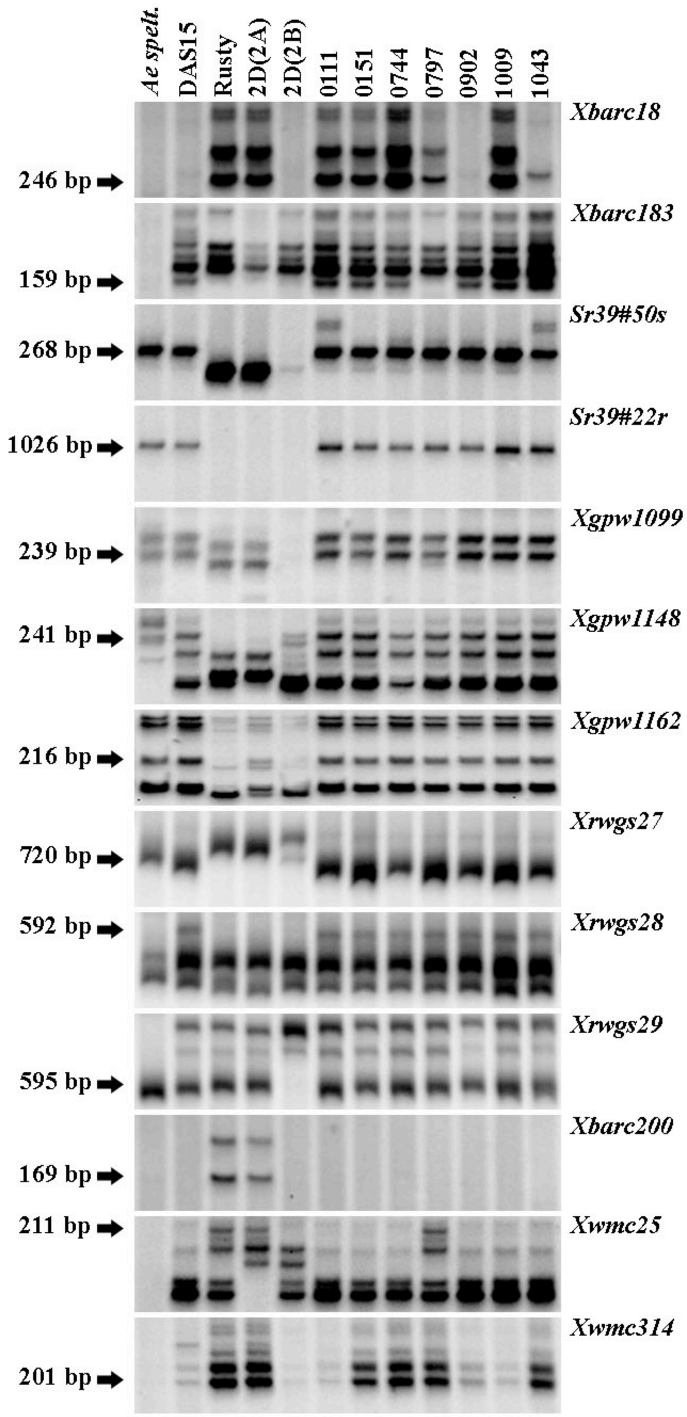

Figure S6 Seven homozgyous IT 2 lines tested with 13 molecular markers that locate to wheat chromosome arm 2BS. *Ae. spelt.* is *Aegilops speltoides* accession PI 369590, the parental line of DAS15. Marker *Xrwgs29* produced a 595 bp band located to wheat chromosome 2B that was either monomorphic or could not be differentiated under mini-gel electrophoretic conditions.
