# Supplementary material for: Introgression and Characterization of a Goatgrass Gene for a High Level of Resistance to Ug99 Stem Rust in Tetraploid Wheat
Source: G3 (Bethesda). 2012 Jun 1;2(6):665–73. doi: 10.1534/g3.112.002386 (PMC3362296; doi:10.1534/g3.112.002386)
Supplement: Supporting Information [file supp_2.6.665_TableS1.pdf]

Table S1 Fragment sizes of microsatellite (SSR) amplicons used to test for allosyndetic recombination of *Sr47*

		Fragment size (bp) [a]	
SSR Marker	No. of Tests	Rusty	DAS15
Xcfa2278	2	142	152
Xgwm55	1	175	162
Xgwm319	1	193	182
Xwmc474	2	161-163[b]	172-174[b]
Xbarc55	2	154-159[b]	136

[a] Fragment size includes a 19-bp M13 primer tail

[b] Variable fragment sizes were due to minor differences observed in some check lines
