# Supplementary material for: Introgression and Characterization of a Goatgrass Gene for a High Level of Resistance to Ug99 Stem Rust in Tetraploid Wheat
Source: G3 (Bethesda). 2012 Jun 1;2(6):665–73. doi: 10.1534/g3.112.002386 (PMC3362296; doi:10.1534/g3.112.002386)
Supplement: Supporting Information [file supp_2.6.665_TableS2.pdf]

Table S2 Allosyndetic recombinants in the BC_2F_1 generation of Rusty/3/Rusty 5D(5B)/DAS15//47-1 5D(5B) classified for nine SSR markers and for plant vigor and fertility [a]

	Stem Rust IT	Plant No.	*Xgpw 4043*	*Xgwm 501*	*Xcfa 2278*	*Xgwm 55*	*Xgwm 319*	*Xwmc 474*	*Xbarc 55*	*Sr39 #22r*	*Xgwm 614*	No. of spikes	No. of seeds
1	0;	0010	S	W	W	W	W	W	W	W	S	8	23
2	0;	0011	S	S	W	W	W	W	W	W	W	5	0
3	0;	0040	S	S	W	W	W	W	W	W	W	8	26
4	0;	0058	W	S	W	W	W	W	W	W	W	11	28
5	0;	0077	S	S	W	W	W	W	W	W	W	8	18
6	0;	0078	S	S	W	W	W	W	W	W	W	4	15
7	0;	0114	S	S	W	W	W	W	W	W	W	5	22
8	0;	0142	S	S	W	W	W	W	W	W	W	7	19
9	0;	0143	S	W	W	W	W	W	W	W	W	7	8
10	0;	0198	S	S	W	W	W	S	S	S	S	11	47
11	0;	0224	S	S	W	W	W	W	W	W	W	6	15
12	0;	0225	S	W	W	W	W	W	W	W	S	8	7
13	0;	0240	S	S	W	W	W	W	W	W	W	11	25
14	0;	0307	W	S	W	W	W	W	W	W	W	10	18
15	0;	0364	W	S	W	W	W	W	W	W	W	7	15
16	0;	0406	W	S	W	W	W	W	W	W	W	10	15
17	0;	0439	S	S	W	W	W	W	W	W	W	14	21
18	0;	0448	S	S	W	W	W	W	W	W	W	7	7
19	0;	0458	S	S	S	S	S	W	W	W	W	3	0
20	0;	0466	S	W	W	W	W	W	W	W	W	6	11
21	0;	0605	S	S	W	W	W	W	?	W	W	11	35
22	0;	0606	S	S	W	W	W	W	W	W	W	15	27
23	0;	0613	S	W	W	W	W	W	W	W	W	11	12
24	0;	0623	S	S	W	W	W	W	W	W	S	8	15

25	0;	0662	S	S	W	S	W	W	?	W	W	5	0
26	0;	0696	S	S	W	W	W	W	W	W	W	15	209
27	0;	0715	S	S	W	W	W	W	W	W	W	10	33
28	0;	0717	S	W	W	?	W	W	W	W	W	6	18
29	0;	0730	S	W	W	W	W	W	W	W	W	4	3
30	0;	0735	S	S	W	W	W	W	W	W	W	3	4
31	0;	0759	S	S	W	W	?	W	W	W	W	11	14
32	0;	0773	S	S	W	W	W	W	W	W	W	4	13
33	0;	0775	S	S	W	W	W	W	W	?	W	5	18
34	0;	0777	W	S	S	S	S	W	W	?	W	11	0
35	0;	0790	S	W	W	W	W	W	W	W	W	10	18
36	0;	0801	W	S	W	W	W	W	W	W	W	10	16
37	0;	0804	S	W	W	W	W	W	W	W	W	6	14
38	0;	0812	S	S	W	W	W	W	W	W	W	7	11
39	0;	0906	S	S	W	W	W	W	W	W	W	16	70
40	0;	0958	S	S	W	W	W	W	W	W	W	10	8
41	0;	0972	S	S	W	W	W	W	W	W	W	12	16
42	0;	1042	S	S	W	W	W	W	W	W	W	6	5
1	2	0111	S	W	W	W	W	W	S	S	S	10	7
2	2	0744	W	W	W	W	W	W	W	S	W	8	10
3	2	0797	S	W	W	W	W	W	W	S	W	3	10
4	2	1002	W	W	W	W	W	S	S	S	S	6	12
5	2	1009	S	W	W	W	W	S	S	S	S	6	13
6	2	0016	W	W	S	S	S	S	S	S	S	9	18
7	2	0120	W	S	S	S	S	S	S	S	S	6	2
8	2	0150	W	W	S	S	S	S	S	S	S	6	2
9	2	0151	W	S	S	S	S	S	S	S	S	8	13

10	2	0242	W	S	S	S	S	S	S	S	S	4	9
11	2	0311	W	S	S	S	S	S	S	S	S	9	0
12	2	0340	W	W	S	S	S	S	S	S	S	9	3
13	2	0361	W	W	S	S	S	S	S	S	S	8	4
14	2	0380	S	W	S	S	S	S	S	S	S	3	6
15	2	0384	W	W	S	S	S	S	S	S	S	6	1
16	2	0387	W	W	S	S	S	S	S	S	S	3	0
17	2	0431	S	S	S	S	S	S	S	S	S	5	9
18	2	0438	W	W	S	S	S	S	S	S	S	5	0
19	2	0556	S	W	S	S	S	S	S	S	S	9	0
20	2	0608	W	W	S	S	S	S	S	S	S	3	1
21	2	0615	W	W	S	S	S	S	S	S	W	9	5
22	2	0679	W	W	S	S	S	S	S	S	S	7	4
23	2	0866	W	W	S	S	S	S	S	S	S	3	7
24	2	0884	W	S	S	S	S	S	S	S	S	2	1
25	2	0901	W	W	S	S	S	S	S	S	S	3	7
26	2	0902	S	W	S	S	S	S	S	S	W	5	12
27	2	0974	W	W	S	S	S	S	S	S	S	13	3
28	2	0975	W	W	S	S	S	S	S	S	S	7	7
29	2	0979	W	W	S	S	S	S	S	S	S	8	9
30	2	0989	W	W	S	S	S	S	S	S	S	9	2
31	2	1014	W	W	S	S	S	S	S	S	S	2	3
32	2	1041	W	W	S	S	S	S	S	S	S	5	2
33	2	1043	W	S	S	S	S	S	S	S	W	3	5
34	2	1051	S	W	S	S	S	S	S	S	W	7	3
35	2	1105	W	?	S	S	S	S	S	S	S	4	1
36	2	1138	W	?	S	S	S	S	S	S	S	6	0

37	2	1174	W	W	S	S	S	S	S	S	S	7	2
1	34	0414	S	S	S	S	S	S	W	W	S	5	6
2	34	0946	W	S	S	S	S	S	W	W	W	1	1

[a] The plants not listed in this table were 191 stem rust susceptible that retained wheat chromatin for the five markers studied by capillary electrophoresis, and 814 IT 0; plants that retained *Ae. speltoides* chromatin for the five markers studied by capillary electrophoresis

W = wheat allele, S = *Ae. speltoides* allele, question mark (?) indicates that the plant could not be scored for that marker

In summarizing this data for Table 1, markers scored as question mark (?) were assigned the value of the proximal and distal markers if they were identical. For example, plant 0605 was assigned W for *Xbarc55* because *Xwmc474* and *Sr39#22r* had both been scored as W
