# Supplementary material for: Introgression and Characterization of a Goatgrass Gene for a High Level of Resistance to Ug99 Stem Rust in Tetraploid Wheat
Source: G3 (Bethesda). 2012 Jun 1;2(6):665–73. doi: 10.1534/g3.112.002386 (PMC3362296; doi:10.1534/g3.112.002386)
Supplement: Supporting Information [file supp_2.6.665_TableS3.pdf]

Table S3 Segregation for rust resistance and marker alleles among progeny of heterozygous plants of five allosyndetic recombinant lines

						χ^2 (1:2:1)		Freq.
Line	IT	Marker	HR	HetR	S	value	*p* (1:2:1)	HR
RWG 35 (0406)	0;	*Xgwm501* & *Xgwm47*	25	40	14	3.1	0.215	0.316
RWG 36 (0696)	0;	*Xgpw4043*	39	39	8	23.1	<0.001	0.453
RWG 37 (0717)	0;	*Xgpw4043*	27	38	8	10.0	0.007	0.370
RWG 38 (0744)	2	*Sr39#50s*	3	24	33	32.4	<0.001	0.050
RWG 39 (0797)	2	*Sr39#50s*	3	51	33	23.3	<0.001	0.034

HR = Homozygous resistant, HetR = Heterozygous resistant, S = Homozygous susceptible. Stem rust resistant plants were classified as homozygous or heterozygous based on molecular marker analysis
